# Brain-computer interface technology for motor rehabilitation in severe stroke: a narrative review

**DOI:** 10.3389/fbioe.2026.1822784

**Published:** 2026-05-04

**Authors:** Yuting Li, Renhui Yi, Zheng Hu

**Affiliations:** 1 Department of Rehabilitation Medicine, Ganzhou People’s Hospital, Ganzhou, Jiangxi, China; 2 Department of Neurosurgery, The First Affiliated Hospital of Gannan Medical University, Ganzhou, Jiangxi, China

**Keywords:** brain-computer interface, motor recovery, neuroplasticity, neurorehabilitation technology, stroke rehabilitation

## Abstract

This review examines the application of brain–computer interface (BCI) technology for motor rehabilitation in patients with severe stroke—a population often excluded from conventional therapies due to minimal movement. BCIs establish electronic links between the brain and external devices, enabling motor intention recognition without muscular activity. By pairing neural activation with sensory feedback, these systems promote neuroplasticity and strengthen adaptive motor pathways. Compared with standard therapies, preliminary evidence suggests BCI interventions may facilitate additional motor recovery, though current effect size estimates are limited by small sample sizes, high study heterogeneity, and inherent performance biases. Effective modalities include motor imagery with functional electrical stimulation, robotic-assisted training in virtual environments, and multimodal systems. Despite promising results, challenges persist regarding signal reliability, protocol optimization, patient selection, and cost. Emerging research focuses on integrating artificial intelligence, adaptive closed-loop systems, and portable platforms to enhance clinical feasibility. Interdisciplinary collaboration may help transition BCI technology from experimental use to routine rehabilitation, improving outcomes for severely impaired stroke survivors.

## Highlights

- Rehabilitation with brain–computer interface and upper limb motor function in ischemic stroke: a randomized controlled trial.
- Contralaterally controlled functional electrical stimulation versus neuromuscular electrical stimulation for recovery of hand function in severe hemiplegia: a randomized controlled trial.
- A comparison of immersive and non-immersive virtual reality-based brain–computer interface for post-stroke motor rehabilitation.
- Brain-computer interface-based soft robotic glove rehabilitation for stroke.
- Brain–computer interface treatment for motor rehabilitation of upper extremity of stroke patients—A feasibility study.
- Brain functional networks study of subacute stroke patients with upper limb dysfunction after comprehensive rehabilitation including BCI training.
- Combined rTMS and virtual reality brain–computer interface training for motor recovery after stroke.

## 1 Introduction

Stroke remains one of the most significant causes of disability worldwide, affecting approximately 15 million individuals each year and resulting in over two-thirds of survivors experiencing chronic motor impairments (Kruse et al., 2020; Angerhöfer et al., 2021). The motor deficits, frequently referred to as post-stroke spasticity, are among the most common sequelae of stroke and, in some instances, alongside the loss of cognitive functions, the health-related quality of life (HRQoL) of the patients is severely compromised, creating a substantial socio-economic burden on healthcare systems (Juckett et al., 2020). A particularly disabling outcome is severe upper limb paresis—operationally defined in this review as a Fugl-Meyer Assessment-Upper Extremity (FMA-UE) score of less than 20, or the complete clinical absence of active wrist and finger extension. This profound functional inactivity is inadequately addressed by most conventional rehabilitation techniques (Baniqued et al., 2021; Mansour et al., 2022).

Most patients with hemiplegia receive treatment through physical therapy-based rehabilitation programmes such as constraint-induced movement therapy and traditional occupational therapy. While these approaches are successful and effective strategies for patients with mild to moderate impairments, they do not meet the needs of those with more severe motor disabilities (Bai et al., 2020; Comino-Suárez et al., 2021). Most conventional methods rely on some degree of voluntary movement and residual function to motivate therapeutic activities, effectively excluding the most severely disabled patients who cannot demonstrate any voluntary movement (Angerhöfer et al., 2021; Remsik et al., 2016). Additionally, many traditional rehabilitation programmes do not implement the intensity and repetition required to facilitate the neuroplastic changes essential for recovery in the more affected individuals (Mane et al., 2020; Zhang et al., 2024). This has led to the development of new neurorehabilitation approaches designed to address these limitations (Bai et al., 2020; Remsik et al., 2016).

The rehabilitation of severe stroke-related motor impairments includes brain–computer interface (BCIs) as one of the treatment options available (Kruse et al., 2020; Mansour et al., 2022; Nojima et al., 2022). Through the use of BCIs, patients can control assistive devices and receive neurofeedback at will through signals—all without muscular activation—due to the connection established between the brain and external devices (Baniqued et al., 2021; Abiri et al., 2019). Patients with severe impairments who lack the capacity to participate in traditional therapy aimed at movement can benefit from this technology. BCIs have the potential to facilitate a form of movement neuroplasticity, whereby deploying motor commands for neural signals coupled with appropriate feedback may enable recovery, even when no observable motion occurs (Mane et al., 2020; Nojima et al., 2022; Rathee et al., 2019).

As evidenced by randomised controlled trials and meta-analyses, BCI technology offers therapeutic potential for stroke rehabilitation (Bai et al., 2020; Mansour et al., 2022; Li et al., 2025; Yang et al., 2022). Recent studies demonstrate that in comparison to conventional therapies, motor function recovery is significantly enhanced with BCI-based interventions in stroke patients, with effect sizes ranging from moderate to large (Yang et al., 2022; Qu et al., 2024; Xie et al., 2022). Improvements have been observed in the functioning of both upper and lower limbs, indicating the possibility of differing profiles of disabilities (Kruse et al., 2020). Furthermore, BCI interventions are advantageous in the chronic and subacute stages of stroke, suggesting their utility throughout the recovery continuum (Yang et al., 2022; Wang et al., 2024).

Technologies such as motor imagery systems (Zhang et al., 2024; Nojima et al., 2022), functional electrical stimulation (Baniqued et al., 2021; Li et al., 2025; Knutson et al., 2016), robotic assistance (Baniqued et al., 2021; Cheng et al., 2020), virtual reality environments (Elashmawi et al., 2024; Johnson et al., 2018), and various others, including multimodal integrations, utilise BCIs and have been explored for stroke rehabilitation (Kwon et al., 2020; Orban et al., 2022). Different therapies have shown promise for particular patient subsets and rehabilitation goals and have demonstrated great potential for their targeted population (Li et al., 2025; Xie et al., 2022). Moreover, the customisability of BCI systems according to the specific needs of each individual patient could enhance productivity and engagement, as well as therapeutic outcomes, for the patient (Rathee et al., 2019; Mane et al., 2019).

Despite the encouraging results, there remains a gap between the implementation of BCI technologies and clinical practice, which must be addressed (Chen et al., 2019; Pillette et al., 2020). Barriers such as signal reliability, ease of use, and affordability all fall under the umbrella of technical limitations and present a significant challenge for universal adoption (Nijboer et al., 2015; Jochumsen et al., 2020). Other issues, such as ideal treatment procedure frameworks, criteria for patients, and efficiency over extended timeframes, also need to be resolved (Mattia et al., 2020). Overcoming these obstacles will be crucial if we wish to maximise the benefits of BCI technologies for stroke rehabilitation (Joshi et al., 2022). Intended audience and scope. This review is written primarily for clinicians and allied health professionals involved in stroke rehabilitation (e.g., rehabilitation physicians, physiotherapists, occupational therapists, and nurses) who seek an accessible synthesis of how BCI systems work, what clinical evidence supports their use in severe motor impairment, and what practical barriers currently limit adoption. Technical concepts are introduced only to the extent needed to interpret clinical feasibility and therapeutic mechanisms.

This review aims to integrate existing information on BCI technology for motor rehabilitation in severe strokes by analysing the fundamentals, system types, clinical evidence, implementation challenges, and future prospects. By thoroughly examining this rapidly developing field, we hope to alert researchers, clinicians, and industry innovators to the potential of BCI systems in transforming rehabilitation for profoundly affected stroke survivors (Mattia et al., 2020; Joshi et al., 2022).

## 2 Fundamental principles and neuroscientific basis of BCI technology

### 2.1 Definition and classification of brain-computer interfaces

BCIs create a communication pathway between the brain and peripheral devices, enabling command execution that bypasses traditional neuromuscular systems (Abiri et al., 2019). These systems capture and process brain activity, translating it into commands for providing feedback or controlling devices, thus holding great promise for restoring functions in people with certain neurological impairments post-stroke (Kruse et al., 2020; Baniqued et al., 2021). BCIs are categorised into different types based on their purpose, application, level of invasiveness, and method of operation. Based on the signal acquisition techniques used, BCIs are considered to be either invasive (using intracortical electrodes or electrocorticography) or non-invasive (using electroencephalography, functional near-infrared spectroscopy, or magnetoencephalography) (Abiri et al., 2019; Orban et al., 2022). BCIs are also categorised based on how they operate as active systems (requiring intent), reactive (responding to external triggers), or passive (monitoring user states without intention) (Abiri et al., 2019).

In the context of rehabilitation, BCIs perform different roles: restorative BCIs seek to increase neural recovery by actively promoting neuroplasticity and reorganisation, potentially aiding users to regain voluntary motor control; while assistive BCIs help overcome lost function by providing different mechanisms for control (Remsik et al., 2016; Soni et al., 2024). For stroke patients, restorative approaches are especially important because they directly target the cerebromotor control malfunction rather than just bypassing it (Mattia et al., 2020).

### 2.2 Brain signal acquisition techniques: invasive and non-invasive

Non-invasive techniques remain dominant for applications in stroke rehabilitation because of their safety and ease of use (Baniqued et al., 2021; Mansour et al., 2022). EEG continues to be the most common form of use. EEG offers great temporal resolution, is portable, and has a low cost (Abiri et al., 2019; Kwon et al., 2020). There are, however, many issues with EEG’s spatial resolution, its susceptibility to noise, and limited access to structures within the deep brain (Orban et al., 2022; Nijboer et al., 2015). For practical purposes, a compromise needs to be made between the number of EEG electrodes and wearability. Most rehabilitation systems use 8–32 channel configurations which help make wearing the device clinically feasible while still providing good quality signals (Nijboer et al., 2015; Jochumsen et al., 2020).

Other non-invasive methods include functional near infrared spectroscopy or fNIRS, which measures haemodynamics while being less susceptible to motion artefacts (Kwon et al., 2020; Orban et al., 2022), and magnetoencephalography or MEG, which supplies excellent spatiotemporal resolution but is limited due to accessibility, cost, and need for additional infrastructure (Abiri et al., 2019). Systems that combine elements of EEG with fNIRS are emerging as promising solutions that utilise fNIRS’ advantages while trying to address its drawbacks and *vice versa* (Kwon et al., 2020; Orban et al., 2022). fNIRS and EEG can work together to capture electrical signals and haemodynamic features which increases the ability to classify signals and overcome challenges associated with single-modality systems (Kwon et al., 2020).

Although offering superior specificity, signal quality, and access to deeper brain structures, invasive approaches are less frequently used in stroke rehabilitation due to the risks of surgery, ethical dilemmas, and other practical constraints (Abiri et al., 2019; Orban et al., 2022). Nonetheless, improvements in the design of chronic implants and other minimally invasive technologies may broaden the use of these techniques in more severe cases where non-invasive techniques are inadequate (Abiri et al., 2019).

### 2.3 Signal processing and feature extraction methods

From a rehabilitation perspective, BCI “signal processing” can be understood as the set of steps that transform raw brain recordings into a reliable decision such as “the patient is attempting to open the hand.” These steps matter clinically because they influence accuracy, setup time, session-to-session consistency, and how much technical support is required (Abiri et al., 2019). Usually, these pipelines consist of preprocessing steps (filtering and artifact removal), feature extraction, and classification (Abiri et al., 2019; Orban et al., 2022). In most cases, signal space containing relevant neural activity is enhanced through spatial filtering such as Common Spatial Patterns (a method that highlights EEG activity most related to the intended movement), temporal filtering, and artifact rejection which all improve the quality of the signal (Abiri et al., 2019; Soni et al., 2024).

Characteristic features extraction concentrates on distinguishing particular signal features, especially those pertaining to sensorimotor rhythms such as alpha (8–13 Hz) and beta (13–30 Hz) event-related desynchronisation/synchronisation (Zhang et al., 2024; Soni et al., 2024) During motor imagery and motor execution, these frequency bands exhibit prominent changes and serve as reliable markers for BCI systems as well (Zhang et al., 2024). Classification is the last stage in which features are transformed to commands and is mostly done using different types of linear discriminant analysis, support vector machines, and more recently deep learning (a data-driven approach that can learn patterns directly from signals but may be harder to interpret clinically) systems (Orban et al., 2022).

As indicated in sources (Soni et al., 2024; Joshi et al., 2022), the historical limitations of BCI dependability have been alleviated due to machine learning and artificial intelligence advancements, which contemporaneously improved classification accuracy and reliability. Improvements in system adaptability and user experience were achieved through deep learning methods, which have excelled at processing multidimensional, non-linear, and non-stationary (meaning the EEG patterns can change across sessions due to fatigue, attention, or recovery) brain signals, as referenced in (Soni et al., 2024). Also gaining popularity are adaptive algorithms, which fine-tune parameters based on performance, to account for increasing variability in signals due to user advancement (Mehrpour et al., 2024; He et al., 2014). Addressing a significant challenge in BCI clinical application reliant on real-time feedback and recalibrated user interaction, novel transfer learning techniques are being designed to reduce the time-intensive calibration and generalisation tasks over multiple sessions and for different users (Joshi et al., 2022; Mehrpour et al., 2024).

### 2.4 Neuroplasticity principles and theoretical foundations of BCI rehabilitation

The therapeutic potential for BCI technology in the rehabilitation of strokes relies fundamentally on neuroplasticity principles. Understanding how BCIs may aid in the neural reorganization hinges on the critical theoretical principle of Hebbian theory which states: “neurons that fire together, wire together.” BCIs enhance conditions for Hebbian plasticity by establishing temporal contingency between motor intention (neural signals) and sensory feedback (visual, tactile, or proprioceptive).

**FIGURE 1:**
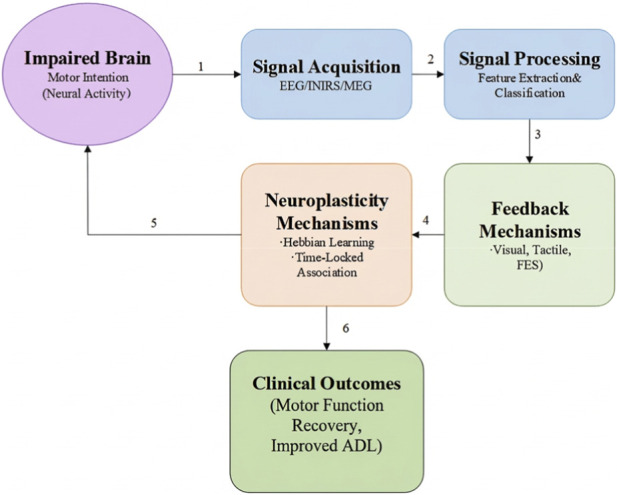
One-page BCI neuroplasticity loop (intention → detection → feedback → Hebbian pairing).

This temporally contingent association of intention and feedback is posited to strengthen synaptic connections resulting in enhanced functional reorganization of the damaged neural circuits. The neuroplastic effect is optimally achieved when there is a delayed reaction time between the motor intention and the sensed action to be executed (feedback) within 200 milliseconds. In addition to employing these principles, BCIs capitalise on experience-dependent plasticity by enabling hyper-repetitive practice of targeted tasks known to drive positive adaptations to the physiology of the brain. BCIs highlight the closed-loop systems and the mechanisms of action these systems may use as depicted in Figure 1 illustrating the framework of BCI-mediated neuroplasticity in stroke rehabilitation.

### 2.5 Neural reorganization after stroke and timing of BCI intervention

The patterns of neural reorganisation after stroke, as well as the recovery progress, present both an opportunity and a challenge for rehabilitation interventions. The recovery undergoes complex reorganisational changes throughout the periods post-stroke (Mane et al., 2020; Carino-Escobar et al., 2019; Wu et al., 2020). In the acute phase, or the period lasting 2 weeks post-stroke, the spontaneous recovery process, dominated by diaschisis resolution, shows promise for therapeutic windows. Unfortunately, practical constraints often limit BCI application during this timeframe (Bai et al., 2020; Wu et al., 2020). Roughly 2 weeks to 6 months post-stroke is known as the subacute phase. This phase marks the period of acquisition for neuroplasticity, dominated by dendritic remodelling, axonal sprouting, and synaptic reorganisation—making it the optimal phase for BCI intervention (Li et al., 2025; Yang et al., 2022). Research suggests that functional recovery is best achieved when BCI interventions are initiated during the 1–3 months phase, likely due to synergistic effects with spontaneous recovery (Li et al., 2025; Yang et al., 2022).

Recovery usually plateaus during the chronic phase (>6 months) as spontaneous neurobiological recovery diminishes (Angerhöfer et al., 2021; Yang et al., 2022). Recently conducted meta-analyses have shown substantial BCI efficacy in both subacute and chronic phases, with some indicative evidence postulating potentially larger effect sizes in subacute patients than in chronic patients (Li et al., 2025; Yang et al., 2022). Recovery predictors, also known as neurophysiological biomarkers, are increasingly derived from motor-evoked potentials, interhemispheric inhibition, and certain measures of connectivity (Mane et al., 2019; Wu et al., 2020). Remaining motor-evoked potentials and certain measures of functional connectivity have recently been shown to help in predicting neuroplastic potential, thus aiding in personalising BCI treatment protocols (Rathee et al., 2019; Mane et al., 2019).

Effective BCI application considers neurophysiological baseline and individual lesion features, as well as preserved neurophysiological baselines, times per plastic windows, and the patient’s residual circuitry (Rathee et al., 2019; Mane et al., 2019). Turning to BCI protocol design, leveraging imaging and biomarker data for personalised BCI protocols aims to define the optimal content and timing of physiotherapeutic interventions to maximise recovery potential, which serves as a boundary shift in guiding BCI protocol design to follow patient needs (Rathee et al., 2019; Mane et al., 2019).

## 3 Major types of BCI rehabilitation systems and their applications

### 3.1 Motor imagery-based BCI systems

Motor imagery (MI) is an essential building block for non-invasive BCI rehabilitation of stroke patients due to the neurophysiological resemblance of imagined and real movements (Zhang et al., 2024). MI-derived BCIs identify sensorimotor rhythm (mostly mu and beta rhythms) changes that occur during the mental rehearsal of motor functions, even when no physical movement takes place (Nojima et al., 2022). This characteristic provides MI-BCIs great utility for severe stroke patients who are unable to move muscles sufficiently to engage in traditional therapies (Zhang et al., 2024; Sebastián-Romagosa et al., 2020).

The clinical effectiveness of MI-BCIs has been validated by several randomised controlled trials (Zhang et al., 2024; Nojima et al., 2022). Zhang et al. (2024) showed that EEG-based motor imagery (MI) training enhances upper limb function recovery compared to purely clinical MI training, with grossly assessed upper limb motor function outcomes also improving. Recent meta-analysis has supported moderate to large effect sizes of MI-BCI application in diverse stroke populations (Nojima et al., 2022; Xie et al., 2022).

As noted in (Zhang et al., 2024; Sebastián-Romagosa et al., 2020), personalised MI approaches, progressive difficulty scaling, as well as multimodal feedback integration, improve therapeutic outcomes, indicating that protocol optimisation is still a topic of research. Notably, MI-BCIs seem to be effective even with patients suffering from chronic strokes (Mansour et al., 2022), which offers opportunities for treatment beyond conventional recovery timelines.

**FIGURE 2:**
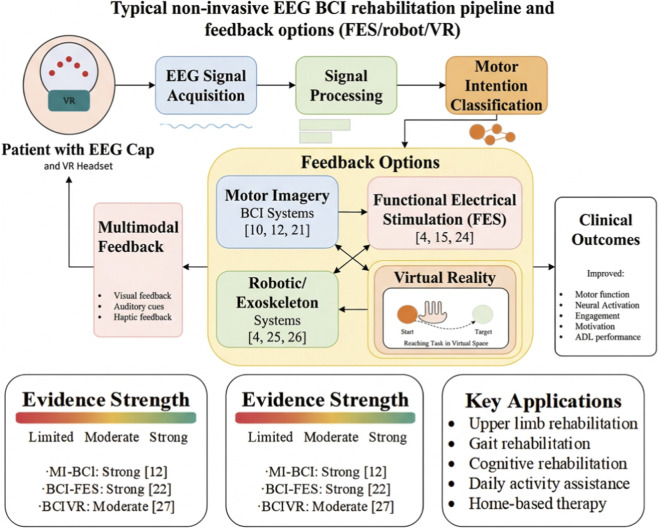
Typical non-invasive EEG BCI rehabilitation pipeline and feedback options.

As illustrated in Figure 2, the system captures EEG signals during imagined movement, processes and classifies these signals, then provides appropriate feedback through visual displays and/or robotic assistance to reinforce the motor intention-feedback loop.

### 3.2 BCI systems combined with functional electrical stimulation (FES)

Combining BCIs with FES is one of the most promising methods for restoring motor abilities in stroke patients with severe impairment. This combination forms a causal relationship between the motor intention detected and the muscle contraction that occurs, which helps strengthen neural networks associated with movement. Usually, BCI-FES systems convert EEG signals corresponding to motor intention through precise temporal patterns into electrical impulses aimed at specific muscle groups; thus, generating completions of the motor tasks with the subsequent reception of naturalistic proprioceptive feedback.

A more recent study by Baniqued et al. showed BCI-robotic systems enhanced upper limb motor functions radically and more than other conventional approaches. Strikingly, these improvements were sustained at 6 months follow-up evaluation, indicating enduring neuroplastic changes. In addition, these authors cited other works where BCI and other rehabilitation methods increased upper limb functionality post-stroke regardless of the rehabilitation approach used.

The contralaterally controlled FES approach is controlled by the patient through voluntary movements of the unaffected hand. This variant is particularly promising, enabling stimulation of corresponding muscles in the affected limb. These techniques were shown by Knutson et al. (2016) and Yang et al. (2022) to enhance hand function and reduce impairment substantially more than conventional therapies with lasting benefits for some time after.

### 3.3 BCI-controlled exoskeleton/robotic assistance systems

The integration of BCI technology with robotic exoskeletons allows for customisable, precise, and coherent assistance with stroke rehabilitation (Baniqued et al., 2021; Cheng et al., 2020). These systems encourage active patient participation by motor intention detection—translated into wearable robotic devices that provide active movement and controlled support (Cheng et al., 2020). The quantifiable performance tracking alongside robotic grade and level precision movement aids in rehabilitation through reproducible movement patterns and graduated assistance (Baniqued et al., 2021; Cheng et al., 2020).

The efficacy of BCI-robot systems has been proven by multiple controlled trials. Moreover, a recent meta-analysis published by Baniqued et al. (2021) confirmed significant improvements and non-BCI robotic therapy provided long-term benefits sustaining chronic strokes.

Continued technological advances improve system functionality. Cheng et al. (2020) created a BCI-controlled soft robotic glove that overcomes the rigid exoskeletons limitation, enabling active hand rehabilitation through easier and more comfortable support. Enhanced therapeutic results alongside increased patient comfort and compliance may stem from advances in wearable soft robotics.

### 3.4 Virtual reality combined with BCI for rehabilitation

The combination of virtual reality (VR) and BCI technologies leads to the development of rehabilitation environments that are energetic, motivating, and immersive, thereby enhancing therapy adherence (Elashmawi et al., 2024; Johnson et al., 2018). Within BCI-VR systems, neural commands are converted to actions in the virtual world, and in return, the user receives multisensory stimulation and therapeutic interactions in the form of games (Elashmawi et al., 2024; Johnson et al., 2018). Such arrangements provide customisation of tasks, an incremental scale of challenge, and realistic validity within the training situation (Elashmawi et al., 2024).

Elashmawi et al. (2024) showed that BCI-equipped VR systems using ML and DL algorithms are capable of producing significant motor gains in stroke patients, thereby improving the effectiveness of rehabilitation. In the same way, Johnson et al. (2018) integrated repetitive transcranial magnetic stimulation with BCI-VR, reporting improved motor recovery relative to standard rehabilitation approaches.

A network meta-analysis comparing immersive versus non-immersive virtual reality for post-stroke upper extremity rehabilitation found that immersive virtual reality produced the greatest improvements in upper extremity function, followed by non-immersive virtual reality systems and then non-immersive gaming consoles, with conventional rehabilitation showing the smallest effects (Hao et al., 2023). VR addresses patients’ motivational needs during extended rehabilitation sessions and prolonged periods of therapy, thus resolving the common challenge of therapy adherence (Johnson et al., 2018).

**FIGURE 3:**
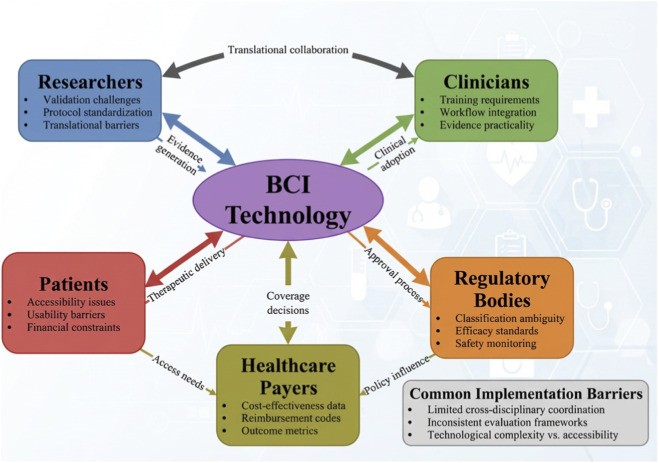
One implementation barriers framework (technical/clinical/accessibility/ethics).

Figure 3 demonstrating the incorporation of EEG signal acquisition, processing, interaction with the virtual environment, and multimodal feedback integration. The system is designed to provide rehabilitation within a motivating virtual environment, in which motor intentions are transformed into actions.

### 3.5 Multimodal hybrid BCI systems

Multimodal hybrid BCI systems are striking innovations in stroke rehabilitation technology, incorporating multiple signal acquisition, feedback, and stimulation mechanisms to increase effectiveness and ease of use. These systems often combine synergistic modalities like EEG with fNIRS, or integrate BCI with other neuromodulation methods such as transcranial direct current stimulation (tDCS).

New comprehensive rehabilitation systems have also been developed. Naros and Gharabaghi (2017) created a multi-layered neuro-robotic interface with real-time EEG control, adaptive assistance, and accurate kinematic feedback, which has shown feasibility and preliminary effectiveness in stroke patients. These multimodal systems may offer solutions for stroke heterogeneity in presentation and enable tailored recovery strategies utilising personalised interventions based on individual deficit profiles and recovery potentials (Joshi et al., 2022; He et al., 2014).

The development of hybrid systems works synergistically with advancements in distributed computing and federated learning techniques that offer enhanced processing while preserving privacy and practical utility (Joshi et al., 2022). This will help close the gap between BCI lab systems and practical clinical use, which would significantly improve rehabilitation for heavily disabled stroke patients, as a result of these advancements (Orban et al., 2022; Joshi et al., 2022).

## 4 Challenges and solutions of BCI technology in stroke rehabilitation

### 4.1 Technical challenges

Progress in BCI technology for stroke rehabilitation holds promise but numerous technical hurdles remain. Everything pertaining to the reliability of the signal remains a foremost concern. The interfaces neurophysiological signals encompass face great challenges from within the body (eye movements and muscle activity), the environment and even changes in one’s mental state or fatigue. These artifacts can hinder accuracy, especially in clinical settings which do not offer controlled lab-like conditions. The problems are exacerbated with the brain’s signals because they are non-stationary. Neuroplasticity along with learning effects alter signal features, which continuously changes, needing constant revision of the system, adaptability.

Issues involving hardware represent some of the greatest obstacles in clinical translation. Imaging EEG systems necessitate a laborious preparatory step which entails positioning numerous electrodes and then checking their impedance, steps that involve a high level of skill likelihood virtually absent in everyday clinical practice. Setup intricacy was found by Jochumsen et al. to greatly influence user satisfaction for patients and caregivers alike. While dry electrode techniques promise relief, they are fraught with their own issues. These methods pose an even greater risk concerning the quality of the signal when compared to wet electrodes. Furthermore, many interface systems suffer from aesthetic issues, along with discomfort for the patients. These hinder therapeutic adherence.

The computational requirements bring along additional difficulties, with sophisticated signal processing algorithms demanding considerable processing capability that is often lacking in clinical environments. There is also the issue of explainability regarding the machine learning methods; although deep learning techniques provide the highest accuracy, validating and evaluating them clinically is severely hindered by the inability to access their inner workings. Technical problems are system-wide, as shown in Table 1, and each has to be addressed individually for progress to be made in clinical use.

**TABLE 1:** Technical challenges and potential solutions in BCI stroke rehabilitation.

System component	Key challenges	Potential solutions
Signal acquisition	• Motion artifacts • Setup complexity • Environmental noise	• Advanced artifact rejection • Dry/quick-apply electrodes • Wearable designs
Signal processing	• Signal non-stationarity • Computational complexity • Personalization needs	• Adaptive algorithms • Edge computing • Transfer learning
Classification	• Inter-subject variability • Low signal-to-noise ratio • Model interpretability	• Subject-specific calibration • Deep learning approaches • Explainable AI methods
Feedback	• Latency • Customization limitations • User engagement	• Low-latency pipelines • Configurable parameters • Gamification elements

Abbreviations: AI, Artificial Intelligence; BCI, Brain-Computer Interface.

As demonstrated in Table 1, addressing these multifaceted technical challenges requires interdisciplinary collaboration among neuroscientists, engineers, and rehabilitation specialists to develop integrated solutions balancing performance with clinical practicality (Abiri et al., 2019; Joshi et al., 2022).

### 4.2 Clinical application challenges

Effective patient selection requires a rigorous neurorehabilitation decision pathway. Clinicians must account for individual determinants beyond mere motor scores; for instance, severe spasticity, global aphasia, profound cognitive deficits, or extensive cortical damage may preclude a patient from actively engaging in motor imagery, thereby severely limiting BCI efficacy. Furthermore, the integrity of the corticospinal tract reserve and the presence of post-stroke pain or hemispatial neglect must be systematically evaluated to determine true candidacy for BCI-mediated therapies.

Integrating There are practical barriers that must be tackled when BCI technology is to be incorporated into the clinical rehabilitation stroke framework, extending beyond the technical aspects. The patient population still poses a challenge because the stroke cohort has considerable heterogeneity in lesion morphology, baseline functional capability, and recovery potential. Xie et al. (2022) was noted that while BCI rehabilitation programmes for the upper extremities range skillfully and broadly, they are moderated by individual features to a great extent. It is possible to predict certain responses because some neurophysiological traits are more likely to yield favourable results. However, standard assessment norms for secondary criteria selection are still proposed to be rudimentary.

Another difficulty is treatment parameter optimisation; the lack of delineated session duration and other criteria, including completion frequency and start progression, creates disunity. Gaps in study protocols complicate the integration of evidence and consequent guidelines formulation. As noted by (Bai et al., 2020), despite positive outcomes in many studies, there tends to be significant inconsistency in the treatment parameters defined. Conventional rehabilitation integration is still disputed, as posed by others. There is dual contention as to whether BCI therapy is better suited as an adjunct to, or a substitute for, conventional approaches, and to what degree those two paradigms could be optimally blended for the greatest effect.

Determining optimal intervention timing represents another challenge, with emerging evidence suggesting differential effects across acute, subacute, and chronic stroke phases. Wang et al. (2024) demonstrated significant benefits of BCI intervention in ischaemic stroke, emphasising the need for effective BCI intervention in relation to the recovery scope and its duration. Limited specialised expertise further complicates implementation, as effective BCI application usually demands multidisciplinary teams that are often lacking in numerous clinical settings.

Addressing the challenges from various stakeholder views simultaneously, as depicted in Figure 3, underlies successful clinical implementation. Translating BCI from the laboratory to clinical practice faces multifaceted systemic barriers. Firstly, standardizing rehabilitation protocols remains a primary hurdle; currently, there is no consensus on optimal session dosage, frequency, or the precise timing of intervention across different stroke stages. Secondly, regulatory frameworks frequently struggle to classify BCI systems—often caught in the gray area between medical devices and investigational tools—which significantly delays clinical approval and complicates insurance reimbursement and cost-effectiveness analyses. Long-term patient adherence also presents a challenge, as the repetitive nature of BCI training can lead to fatigue or demotivation. Finally, the ethical implications of BCI must be systematically addressed. These include concerns regarding data privacy, ensuring informed consent in patients with cognitive or communication impairments, and carefully weighing the risk-benefit ratio of invasive versus non-invasive systems. These translational issues require rigorous interdisciplinary oversight before widespread clinical adoption can be realized.

Translating BCI from laboratory to clinic faces multifaceted systemic barriers. Standardizing rehabilitation protocols remains a primary hurdle; currently, there is no consensus on optimal session dosage, frequency, or the precise timing of intervention across acute, subacute, and chronic phases. Furthermore, regulatory frameworks frequently struggle to classify BCI systems—often caught in the gray area between medical devices and investigational tools—which delays clinical approval and complicates insurance reimbursement and cost-effectiveness analyses. Additionally, the ethical implications of BCI, particularly concerning data privacy, informed consent in cognitively impaired patients, and the equitable accessibility of these expensive technologies, require rigorous interdisciplinary oversight before widespread adoption can be realized.

### 4.3 Accessibility and dissemination challenges

Research on BCI rehabilitation technology has noted its potential benefits; however, its widespread accessibility is still lacking. Economic factors serve as the foremost barrier to distribution, as extensive systems require significant financial resources for obtaining equipment, purchasing software licenses, and modifying existing infrastructure (Chen et al., 2019; Jochumsen et al., 2020). Resource-constrained areas are extremely limited in regard to these resources, as identified by (Chen et al., 2019), where cost-effectiveness is vital. Maintenance, technical support, and training for the BCI systems also incur ongoing operational expenses, providing additional economic difficulties (Juckett et al., 2020). Specialized academic centres, which focus on developing new technologies, are further exacerbated by the lack of community hospital and rural patient access, concentrating and limiting the use of these BCIs. Patients are left with the primary burden from the underdeveloped insurance coverage and reimbursement structures within the system (Chen et al., 2019).

Additional barriers stem from insufficient workforce and personnel dedicated to rehabilitation medicine, developing engineering, and computer science, which are integral to the effective application of these technologies (Juckett et al., 2020; Baniqued et al., 2021). These gaps, alongside the limited exposure rehabilitation practitioners receive to advanced neurotechnologies, exacerbate the knowledge gap among frontliners (Juckett et al., 2020). Technical personnel dedicated to setting up and troubleshooting these sophisticated systems are often lacking within rehabilitation—aggravating the resource problem within the field (Vinoj et al., 2019).

The accessibility problems are made even more complicated by the technological intricacy of traditional BCI systems, as many platforms operate within specialised environments and require specialised skills (Jochumsen et al., 2020). As highlighted in Table 2, new portable systems have a marked advantage over conventional laboratory-based equipment in critical accessibility factors required for clinical implementation.

**TABLE 2:** Comparison of conventional and portable BCI systems for clinical rehabilitation.

Parameter	Conventional laboratory systems	Emerging portable systems
Signal quality	High-density with excellent resolution	Fewer channels with adequate resolution
Setup time	30–60 min with technical assistance	5–15 min, potentially self-applicable
Equipment cost	$10,000-$100,000+	$1,000-$10,000
Mobility	Limited, typically fixed installations	High, suitable for diverse settings
Technical expertise	Extensive (EEG technicians, engineers)	Minimal to moderate (trained therapists)
User interface	Complex, research-oriented	Simplified, clinically-oriented
Maintenance	Regular technical maintenance	Minimal, user-serviceable components

Abbreviations: BCI, Brain-Computer Interface; EEG, Electroencephalography.

As shown in Table 2, portable BCI systems offer advantages in accessibility dimensions despite certain technical compromises (Chen et al., 2019; Nijboer et al., 2015). The evolution toward more accessible systems represents a crucial development for expanding BCI rehabilitation beyond specialized centers to diverse clinical environments (Baniqued et al., 2021; He et al., 2014; Nijboer et al., 2015) documented substantial differences in user experience across BCI systems—factors directly impacting clinical utility. Addressing the accessibility gap requires developing technologies balancing effectiveness with practical implementation considerations while ensuring affordability and usability for broader adoption.

## 5 Emerging trends and future perspectives of BCI technology

### 5.1 Applications of artificial intelligence and deep learning in BCI

The infusion of artificial intelligence (AI) and deep learning techniques is revolutionary in the context of BCI technology for stroke rehabilitation. Most BCI systems in the past relied heavily on conventional paradigms of machine learning, such as linear discriminant analysis and support vector machines. These approaches are feature-centric, require considerable engineering, and often struggle with the highly variable and non-stationary neurophysiological signals (Abiri et al., 2019; Soni et al., 2024). With the advent of deep learning, features are no longer burdensome to construct as a multitude of deep learning architectures with greater capabilities are emerging. Convolutional neural networks, recurrent neural networks, and even transformers have the ability to hierarchically extract features from raw brain signals, alleviating the burden posed by traditional methods to some extent (Joshi et al., 2022; Soni et al., 2024). Most importantly, AVEs outperform other techniques in the accuracy of motor imagery pattern classification, an essential function for stroke rehabilitation using BCI (Soni et al., 2024). Recently, the development of explainable AI (XAI) has started trying to solve the “black box” problem that has, up to this point, prevented the clinical use of deep learning technologies (Joshi et al., 2022; Soni et al., 2024). Such methods bolster clinician trust and aid in regulatory processes by offering interpretable visual explanations of image and classification decisions. In addition, transfer learning paradigms appear to be especially well-suited for overcoming the data scarcity problems typical of clinical BCI applications (Joshi et al., 2022; Mehrpour et al., 2024). The ability to draw insights from larger datasets or from related tasks is capable of decreasing the calibration burden and increasing the reliability of the system for individual patients with scant training data (Joshi et al., 2022). Adaptive deep learning frameworks that mould system parameters to user inputs in real time—as described by Soni et al. (2024)—are much more sophisticated than previous models and adapt to neuroplastic changes to rehabilitation systems during rehabilitation. These algorithms adjust to the neuroplastic changes that occur during rehabilitation and enable sustained therapeutic interventions while preserving effective system operation over long periods.

The use of federated learning frameworks in BCI systems integrates a novel strategy for mitigating privacy issues along with model development at different institutions (Joshi et al., 2022). Instead of centralising patient data, federated learning permits models to be trained locally at diverse clinical sites, with only model parameters shared centrally (Joshi et al., 2022). This method described by Joshi et al. (2022) enables the creation of robust and generalisable algorithms while keeping the patients’ data safe, which is extremely important considering the sensitive neurophysiological data. With the advancement of these AI techniques, they stand to improve the technical and clinical operation of BCI rehabilitation systems, which may help close the divide between lab BCI demonstrations and practical clinical use.

### 5.2 Development of closed-loop adaptive BCI systems

The advancement of closed-loop adaptive BCIs transforms stroke rehabilitation technology by integrating systems that respond to tailored patients’ performance, neurophysiological condition, and recovery path (Mane et al., 2020; Rathee et al., 2019; He et al., 2014). Usually, BCI systems operate with static parameters during intervention blocks which could be counterproductive as patients’ capabilities shift over time (Joshi et al., 2022; He et al., 2014). Such frameworks are usually static and work with pre-defined shift parameters. On the other hand, adaptive systems retain static parameters during intervention blocks which is counterproductive due to the lack of dynamic engagement. Adaptive systems, however, track performance metrics and neural indicators in real time, modifying stimulation parameters, level of difficulty, and feedback mechanisms in order to optimise therapeutic engagement to maximise neuroplastic responses (Rathee et al., 2019; He et al., 2014).

Mane et al. (2020) showcased the effectiveness of a brain state-dependent peripheral stimulation methodology where motor recovery is enhanced with greater peripheral stimulation applied at optimal postural control phases compared to standard protocols. This closed-loop precision enables optimal conditions for Hebbian plasticity where a consistent association between action and feedback strengthens the connections formed during motor intention (Mane et al., 2020; Rathee et al., 2019). In a similar system (He et al., 2014), created an integrated neuro-robotic interface with real-time EEG signal-driven support level modulation for rehabilitation which maintains the appropriate level of challenge throughout each session. Alleviating frustration due to excessive difficulty or lack of challenge helps mitigate disengagement during therapy, which is paramount for therapeutic participation (Rathee et al., 2019; He et al., 2014).

As noted in sources (He et al., 2014; Rathee et al., 2019), advanced closed-loop systems are integrating other patient physiological signals apart from EEG such as electromyography, kinematics, and even autonomic measures, to refine the definition of patient status and improve the tailoring of intervention parameters. More sophisticated interventions can be applied to address specific needs or even prevent these issues before the patient experiences fatigue, frustration, or disengagement (Rathee et al., 2019; Joshi et al., 2022).

Aadaptive BCI systems incorporate multiple feedback loops with varying timescales, including real-time adjustments of signal processing at the millisecond level and more protocol-level changes aligned with recovery trajectories over longer durations. This multi-tiered adaptation approach alongside stroke patients' evolving ability facilitates optimised engagement during rehabilitation.

### 5.3 Advances in wearable and portable device development

The shrinking size and increased portability of BCI hardware marks a pivotal development in the evolution of rehabilitation technologies, enabling access beyond specialised research settings to clinical environments and home-based applications (Chen et al., 2019; Nijboer et al., 2015; Jochumsen et al., 2020). BCI systems, as they are classically designed, incorporate cumbersome equipment and require controlled environments as well as technicians with high levels of expertise, which has limited the use of BCIs in clinical practice (Chen et al., 2019; Jochumsen et al., 2020). The most recent breakthroughs in electrode technology, alongside other components such as signal acquisition hardware and processing algorithms, have made it possible to assemble more compact and user-friendly systems that can be deployed in real-world settings (Nijboer et al., 2015; Jochumsen et al., 2020).

Innovations in dry electrode technology have greatly simplified setup, as shown in the systems studied by (Nijboer et al., 2015), which do not require gels or complex skin preparation and have proven to acceptably capture signal quality. Although these electrodes tend to have higher resistance and are more prone to motion artefacts than traditional wet electrodes, advanced signal processing algorithms have increasingly compensated for these shortcomings (Nijboer et al., 2015). In addition, there have been advancements in the miniaturisation of amplifiers and the wireless transmission of data which have resulted in lightweight bulk-free systems that improve patient comfort while enhancing unobstructed natural movement during rehabilitation exercises (Nijboer et al., 2015; Jochumsen et al., 2020).

Wearable BCI systems have proven to be more user-friendly and acceptable for patients and clinicians due to design improvements based on user-centred frameworks (Chen et al., 2019; Jochumsen et al., 2020; Jochumsen et al., 2020) reported that ease of mounting, comfort during prolonged use, and aesthetics were major determinants of engagement with BCI technology. Their assessment of EEG headset mounting systems by patients, therapists, and family members underscored the need for user-friendly design and low setup effort and time for clinical use (Jochumsen et al., 2020). Furthermore (Chen et al., 2019), emphasised the need for dependable technology, including robust battery life and simple operating guides, as paramount to successful remote neurorehabilitation. These advances driven by users’ needs are important steps toward effective BCI integration into rehabilitation in various settings.

### 5.4 Multimodal system integration and personalized approaches

The integration of different neurotechnology methods within cohesive rehabilitation frameworks provides personalised multi-faceted therapies yielding higher treatment outcomes (Kwon et al., 2020; Mehrpour et al., 2024; Naros and Gharabaghi, 2017). Unimodal BCI approaches usually face challenges addressing the intricate and diverse stroke-related impairments despite having demonstrated effectiveness in controlled environments (Kwon et al., 2020; Joshi et al., 2022). Multimodal systems that integrate multiple signal acquisition, stimulation, and feedback mechanisms offer broad scope therapeutic intervention and improved effectiveness for a wider range of patients (Kwon et al., 2020; Orban et al., 2022; Naros and Gharabaghi, 2017).

The integration of EEG and fNIRS as hybrid signal acquisition methods is one of the major breakthroughs in BCI precision and dependability (Kwon et al., 2020; Orban et al., 2022; Kwon et al., 2020) showed the benefits hybrid systems bring from combining electrophysiological and haemodynamic measures where EEG’s high temporal resolution is paired with fNIRS’ superior resistance to motion artifacts. These hybrid systems provide better classification accuracy than single-modality systems even with low channel counts, thus improving clinical applicability (Kwon et al., 2020). Enhanced intent detection made possible with fNIRS enables a much wider range of patients to operate BCI systems (Kwon et al., 2020; Orban et al., 2022).

The combination of BCI with other forms of neuromodulation techniques has been especially promising in improving neuroplasticity and functional recovery (Soni et al., 2024; Naros and Gharabaghi, 2017). For instance (Mehrpour et al., 2024), showed that motor imagery BCI showed better motor improvement when combined with tDCS as compared to tDCS or BCI only, which means some level of tDCS and BCI must have worked together in aiding motor improvement. Also (Naros and Gharabaghi, 2017), demonstrated that beta-band tACS increased the efficacy of BCI training and other forms of BCI training by modulating valued frequency bands as well. In Table 3, the most important multimodal BCI strategies, their stroke rehabilitation, and other relevant benefits are highlighted.

**TABLE 3:** Multimodal BCI approaches for stroke rehabilitation.

Integration type	Component technologies	Potential advantages	Key references
Hybrid signal acquisition	EEG + fNIRS	Improved classification accuracy, reduced noise sensitivity, complementary neural information	Kwon et al. (2020), Orban et al. (2022)
Neuromodulation + BCI	tDCS/tACS + BCI	Enhanced cortical excitability, accelerated learning, targeted neuroplasticity	Mehrpour et al. (2024), Naros and Gharabaghi (2017)
Multiple feedback channels	Visual + Haptic + Auditory	Enriched sensory experience, engagement of multiple neural pathways, accommodation of sensory deficits	Elashmawi et al. (2024), Johnson et al. (2018)
Robot-assisted BCI	BCI + exoskeleton/FES	Precise movement assistance, somatosensory feedback, graduated support	Baniqued et al. (2021), Cheng et al. (2020)
VR-enhanced BCI	BCI + immersive environments	Increased motivation, ecological validity, cognitive engagement	Elashmawi et al. (2024), Johnson et al. (2018)
Comprehensive monitoring	Neural + physiological + kinematic measures	Holistic patient state assessment, early detection of fatigue/frustration, precise progress tracking	Rathee et al. (2019), He et al. (2014)

Abbreviations: BCI, Brain-Computer Interface; EEG, Electroencephalography; FES, Functional Electrical Stimulation; fNIRS, Functional Near-Infrared Spectroscopy; tACS, Transcranial Alternating Current Stimulation; tDCS, Transcranial Direct Current Stimulation; VR, Virtual Reality.

As shown in Table 3, diverse multimodal approaches offer specific advantages for addressing the complex challenges of stroke rehabilitation. The particular combination of technologies can be tailored to individual patient characteristics, impairment profiles, and therapeutic goals, enhancing the personalization of rehabilitation interventions (Orban et al., 2022; Joshi et al., 2022).

### 5.5 Key issues in clinical translation and interdisciplinary collaboration

The conversion of BCI technology from laboratory demonstrations into clinical practice requires decidedly scientific, practical, and systemic difficulties to be solved by synergistic interdisciplinary work (Table 4). Much like multiple other complexities, system performance and usability are two areas that undergo continuous technological enhancements. However, barriers, including protocol standardisation, evidence accumulation, workforce training, and economic factors, are non-technological and unsolvable with engineering alone. Addressing these multifaceted issues requires a collaborative approach. BCI practitioners, rehabilitation specialists, neuroscientists, and implementation scientists must all come into play. Defined assessment protocols alongside measurable outcomes aim to resolve the absence of clear clinical guidelines in evidence synthesis. As discussed by Mattia et al., neurophysiological markers alongside functional improvements, to even self-reported measures must be encompassed in BCI rehabilitation trial outcome assessments. This Promotoer study protocol highlights rigorous evaluative approaches of response determinants that are both immediate and sustain efficacy long-term. Methodologically sound research like this is imperative to gaining evidence for regulatory, clinical, and funding approvals.

**TABLE 4:** Summary of key clinical trials in BCI-mediated stroke rehabilitation.

Study (author, year)	Sample size (N)	Stroke stage	BCI modality/intervention	Control group/comparator	Primary outcome measures	Key clinical findings
Wang et al. (2024)	296	Ischemic stroke	MI-BCI + FES + VR + CT	CT	FMA-UE	Greater FMA-UE improvement (+3.35 mean diff) vs. CT
Knutson et al. (2016)	132	Chronic stroke (6–24 months)	CCFES	cNMES + TOT	BBT, FMA-UE, ARAT	FMA-UE improved significantly more than control; BBT gains comparable
Sebastián-Romagosa et al. (2020)	51	Mostly Chronic (45 chronic, 6 subacute)	MI-BCI + FES + VR	Baseline	FMA-UE, MAS	Significant, long-lasting FMA-UE gains (+4.68) and spasticity reduction
Cheng et al. (2020)	11	Chronic stroke (>6 months)	MI-BCI + SRG	SRG	FMA-UE, ARAT	Sustained functional improvements; elicited vivid kinesthetic experiences
Wu et al. (2020)	25	Subacute stroke (1–6 months)	BCI + Exo + CT	CT	rs-fMRI, FMA-UE, ARAT, WMFT	Superior motor gains; positive reorganization of brain functional networks
Johnson et al. (2018)	3	Chronic stroke (∼1 year)	Real rTMS + VR-BCI	Sham rTMS	BBT, IHI	Reduced IHI; increased ipsilesional activation; enhanced BCI performance

Abbreviations: ARAT, Action Research Arm Test; BBT, Box and Block Test; CCFES, Contralaterally Controlled Functional Electrical Stimulation; cNMES, Cyclic Neuromuscular Electrical Stimulation; CT, Conventional Therapy / Comprehensive Rehabilitation; Exo, Exoskeleton hand; FES, Functional Electrical Stimulation; FMA-UE, Fugl-Meyer Assessment-Upper Extremity; IHI, Inter-hemispheric Inhibition; MAS, Modified Ashworth Scale; MI-BCI, Motor Imagery Brain-Computer Interface; rs-fMRI, Resting-state functional Magnetic Resonance Imaging; rTMS, Repetitive Transcranial Magnetic Stimulation; SRG, Soft Robotic Glove; TOT, Task-Oriented Training; VR, Virtual Reality; WMFT, Wolf Motor Function Test. *Mixed: 45 chronic and 6 subacute stroke patients.

As outlined in references (Juckett et al., 2020; Chen et al., 2019), the workflows of educational programmes supporting development are also significant while conducting successful clinical translation. Juckett et al. (2020) have noted the lack of adequate neurorehabilitation evidence-based practice training of rehabilitation professionals as an important barrier. Tackling the challenge from this perspective requires that BCI be taught as part of the rehabilitation training curriculum, accompanied by other continuing education materials, and offer interdisciplinary clinical-applied and engineering courses (Juckett et al., 2020; Joshi et al., 2022). Also, the creation of neurotechnology tailored implementation frameworks will enable systematic evaluation of context factors concerning BCI controlled healthcare processes and the influence of various controlled processes in different healthcare institutions (Juckett et al., 2020).

Developing and implementing sustainable ecosystems for innovation in BCI requires innovation in collaborative frameworks which go beyond traditional discipline and institutional demarcations (Juckett et al., 2020; Joshi et al., 2022). Partnerships between academia and industry, multi-centre studies, and design with patients are examples of how to accelerate translation and align technological advances with clinical demands and limitations (Chen et al., 2019; Joshi et al., 2022). Also, the involvement of healthcare administrators, policymakers, and payers early in the development phases addresses systemic and economic barriers to widespread integration (Juckett et al., 2020; Joshi et al., 2022). These cooperative strategies recognise that the successful clinical translation of technology in practice is not simply about meeting operational standards, but rather involves intricate relationships among multiple constituents in the healthcare framework.

## 6 Conclusion

Navigating the rehabilitation terrain for severe stroke patients suffering from motor impairments reveals BCI technologies as an invaluable asset. BCIs provide therapeutic avenues for patients who cannot partake in conventional movement-centred therapies. Increasing data demonstrates substantial enhancements in motor function, particularly upper limb rehabilitation, when compared to traditional methods. This is corroborated by meta-analyses showing moderate to large effect sizes across various stroke cohorts. BCI systems take advantage of neuroplasticity principles by means of temporal bonding between detected neural intentions and aligned sensory feedback, thereby invoking favourable conditions for Hebbian plasticity and enabling functional reorganisation, even in chronic stroke patients. The range of BCI modalities—including motor imagery-based systems, functional electrical stimulation, robotics, virtual reality, and other multimodal combinations—form a distinct therapeutic arsenal suitable to varying patient profiles and rehabilitation goals. However, alongside the promising advancements lie formidable translational obstacles, including technical constraints (signal dependability, hardware limitations, computational requirements), practical clinical application (patient criteria, parameter tuning, fusion with traditional therapy), and affordability gaps (high financial burden, sparse specialised skill, complexity of technology). Through enhancements such as artificial intelligence, closed-loop adaptivity, portable BCI design, and multimodal integration approaches, newly emerging trends indicate a welcome shift. The ongoing development of these technologies, along with relevant clinical considerations, could support the shift of BCI-based strategies from niche applications to routine rehabilitation practice. Adoption into clinical practice will require the development of simple, economical systems with proven effectiveness over time. These systems should be readily scalable to meet clinical demand. The primary intent of BCI technology is to markedly improve rehabilitation outcomes for stroke survivors with the most severe disabilities by capitalising on the exceptional plasticity of the human nervous system with precisely focused brain interventions, and in doing so, restore function and independence to those who have very few therapeutic options available.
